# Knowledge Gaps in Understanding the Etiology of Anemia in Indonesian Adolescents

**DOI:** 10.1177/0379572120979241

**Published:** 2021-07-20

**Authors:** Kesso Gabrielle van Zutphen, Klaus Kraemer, Alida Melse-Boonstra

**Affiliations:** 1 4508 Wageningen University & Research, Wageningen, the Netherlands; 2 Sight and Life Foundation, Basel, Switzerland; 3 Johns Hopkins Bloomberg School of Public Health, Baltimore, MD, USA

**Keywords:** anemia, adolescents, etiology, micronutrients, hemoglobinopathies, inflammation

## Abstract

**Background:**

Anemia is a public health problem among adolescents in Indonesia. Strategies to prevent or treat anemia should be tailored to local conditions, taking into account its specific etiology and prevalence in a given setting and population group.

**Objective:**

This review aims to (1) identify and synthesize the current knowledge on the etiology of anemia among adolescents in Indonesia, (2) reveal knowledge gaps in this area, and (3) suggest directions for future research and programmatic work.

**Methods:**

We systematically searched PubMed, Web of Science, Scopus, Medline, and WorldCat databases for peer-reviewed journal articles to identify which etiological factors were related to anemia among Indonesian adolescents. Research papers were reviewed and included in the review according to inclusion criteria.

**Results:**

Of 13 studies, 8 showed that anemia was associated with iron deficiency; 4 are suggestive of vitamin A deficiency; and 2 of folic acid deficiency. Five studies underscore different etiological determinants for anemia, such as malaria, protein and energy malnutrition, vitamin B2 deficiency, calcium, and vitamin C deficiency. Based on these findings, we developed a framework on knowledge gaps on the etiology of anemia among adolescents in Indonesia, divided in 3 levels of knowledge: (1) significant knowledge gaps, (2) knowledge gaps, and (3) established knowledge.

**Conclusions:**

The knowledge gaps around the etiology of anemia among Indonesian adolescents are significant. Our framework emphasizes the need for further research across all etiological factors, namely inadequate nutritional intake and absorption, genetic hemoglobin disorders, infection and inflammation, and menstrual disorders.

## Introduction

According to the 2013 Indonesian National Basic Health Research Survey, approximately 23% and 12%, respectively, of 13- to 18-year-old girls and boys are affected by anemia,^1^ thereby classifying anemia as a moderate public health problem among adolescents in Indonesia. In addition to issues with the coverage of and compliance with iron folic acid (IFA) supplementation programs, an important reason for the apparent failure to reduce the prevalence of anemia is that many programs are designed with the assumption that the only cause of anemia is iron deficiency (ID).^2^ However, projections of prevalence derived from the concentration of hemoglobin (Hb) alone do not allow for this conclusion and ignore the role of other causes.^2^

The etiology of anemia is complex. Biologically, anemia can be classified into 3 processes, namely decreased erythrocyte production, increased erythrocyte destruction, and increased erythrocyte loss. These processes are broadly determined by nutrition, infectious disease, genetics, and heavy menstrual beeding. Although ID is the most common nutritional deficiency leading to anemia, deficiencies in not only folate, vitamin B12, and vitamin A but also B6, C, D, E, riboflavin, copper, and zinc can all contribute to anemia. Environmental enteric dysfunction (EED) and disorders of the small intestine leading to malabsorption can cause anemia indirectly, and infectious diseases and inflammation can impair iron absorption and metabolism or may lead to increased nutrient losses. Hereditary blood conditions, in the form of structural variation or reduced production of the globin chains of Hb, are an important cause of anemia globally. Heavy menstrual bleeding can also lead to increased erythrocyte loss. This article provides an in-depth analysis of the etiology of anemia among adolescents in Indonesia.

## Methods

### Literature Search

We searched databases for empirical articles to identify which etiological factors were related to anemia among adolescents in Indonesia. We did not set a cutoff date, and only articles published in English were considered. The databases included were PubMed, Web of Science, Scopus, Medline, and WorldCat. The search terms (title, abstract, keywords) used were “anemia” OR “anaemia” OR “anemic” OR “anaemic” AND “adolescent” AND “Indonesia.”

### Eligibility Criteria

**Figure 1.**
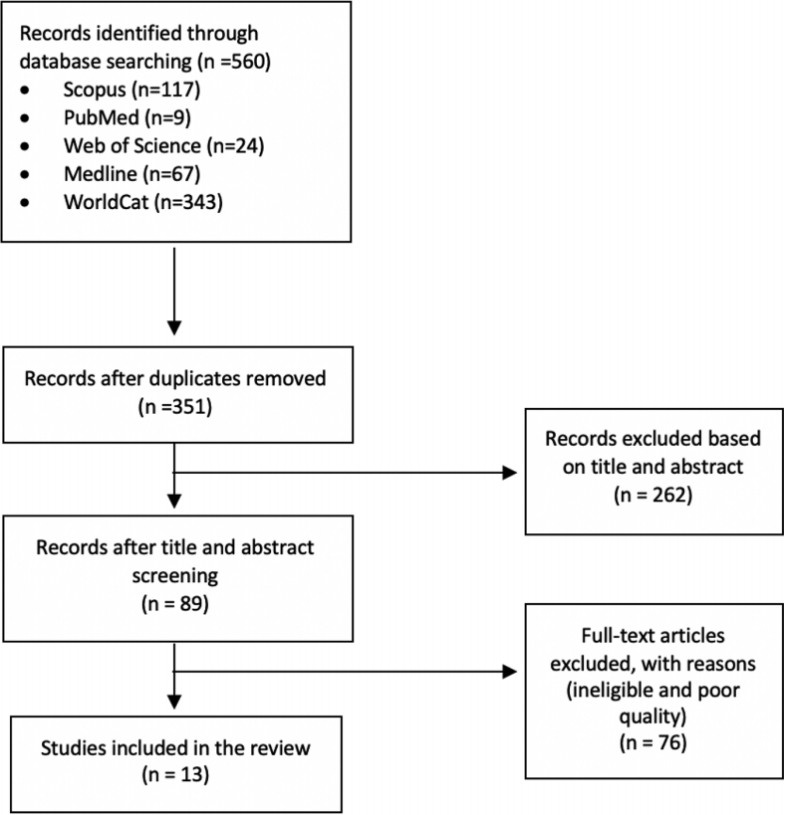
The flow diagram illustrating the selection of studies.

We used the World Health Organization definition of “adolescents” (10-19 years), with the exception of one article which reported data for adolescents aged 10 to 20. Articles were peer-reviewed, with the exception of one high-quality doctoral thesis. Included in this review are articles in which associations between an etiological factor and anemia are reported, including studies in which an etiological factor was used as a covariate, rather than as the primary focus. We excluded single case studies and studies where the sample sizes of disaggregated groups were too small (<10 participants). Thirteen studies fulfilled our inclusion criteria (Figure 1). One doctoral thesis consisted of 4 separate yet related studies. Each study was added as a separate item. No statistical tests were run as part of this review.

## Results

### Overview of Selected Studies

Table 1 provides a detailed summary of the studies with regard to study characteristics as well as anemia prevalence at baseline and lays out their conclusions and remarks. Table 2 lays out the findings of each study with respect to the etiological factors and their relationship with anemia status.

**Table 1.** Summary of Studies Examining Effects of Etiological Determinants of Anemia Among Adolescents in Indonesia (in Ascending Order of Year of Publication)

Reference	Location (urban, rural, or coastal)	Sample size (study design)*Additional information*	In/out of school	Participants (age, sex, pre/post menarche)	Anemia prevalence in population or at baseline (%)	Main etiological determinants assessed^a^	Other etiological determinants mentioned^b^	Deworming and/or inflammation markers measured	Conclusion regarding etiology of anemia	Remarks
Kaetelhut et al (1996)	Jakarta (urban)	84 (RCT) *-Supplementation* *-5 weeks* *-weekly basis* *-vitamin A, C, iron, and extra folate (intervention) vs iron and folic acid (control)*	In school	13-16 years Female Post menarche	3% (population) 29.2% (baseline)	Supplement intake: -Iron -Vitamin A -Vitamin C	NA	Deworming	Iron supplementation, regardless of other nutrients added to the supplement, led to a significant improvement in Hb concentrations. Multiple micronutrient supplementation (vitamin A and C) as compared to iron supplementation alone appears to have some beneficial effects on Hb concentration. ID may be an important factor for anemia as well as other micronutrients.	Lack of statistical significance of multiple micronutrient supplementation as compared to iron supplementation alone on Hb concentrations and anemia prevalence may be due to the too short duration of treatment, low compliance and side effects. Potentially adequate level of micronutrient intake in this middle-income group
Angeles-Agdeppa et al (1997)	East Jakarta (urban)	363 (RCT) *-Supplementation* *-3 months* *Daily:* *-Iron, vitamin A, folic acid, vitamin C* *Weekly:* *-Iron, vitamin A, folic acid, vitamin C (120 mg iron)* *-Iron, vitamin A, folic acid, vitamin C (60 mg iron)* *Placebo*	In school	14-18 years Female Post menarche	21.1% (population)	Supplement intake & status markers: -Iron -Vitamin A	NA	Deworming	The weekly supplement with Fe (low iron content) was most efficient in improving Hb and building iron stores. ID may be an important factor for anemia.	Inflammation not taken into account, so ID may be underestimated; higher dose of iron supplements gives fewer positive results due to side effects; separate effect of vitamin A on Hb concentrations or anemia status cannot be determined from this article, thus cannot conclude that vitamin A deficiency is the cause for anemia.
Soekarjo et al (2001)	Surabaya (urban) Madura (rural)	6486 (cross-sectional)	In school	12-15 years Female and male Pre and post puberty/menarche	20.6% (total) 19.1% (urban) 22.1% (rural) 15.4% (boys) 25.8% (girls)	Dietary intake: -Iron -Vitamin A	Micronutrient deficiencies	None	Girls had a lower chance of being anemic when they had a higher retinol intake and a lower provitamin A intake from plant foods. Boys had a higher risk of anemia when they had lower retinol intake. Low retinol intake and low iron intake are an important determinant for anemia; ID may be another important factor for anemia.	Causation cannot be inferred due to study design; the higher risk of anemia that was associated with a higher vitamin A intake from vegetables might be caused by the high fiber content of vegetables, which inhibits absorption of nonheme iron; the role of iron in anemia was deduced/implied and not statistically observed.
Februhartanty et al (2002)	Kupang, East Nusa Tenggara (urban)	150 (RCT) *-Supplementation* *-16 weeks* *-Placebo (weekly)* *-Iron (weekly)* *-Iron (4 days during menstruation)*	In school	NA; mean age 14.6 ± 1.1 years Female Post menarche	49.6% (baseline)	Supplement and dietary intake: -Iron	-Nutrient absorption enhancers (vitamin C) and inhibitors (calcium)	Deworming	Lack of iron was an underlying factor for low Hb and ferritin levels.	–
Soekarjo et al (2004)	Surabaya (urban) Bangkalan and Sampang (rural)	3616 (RCT) *-Supplementation* *-14 weeks* *-Iron, folate (weekly)* *-vitamin A (weekly)* *-iron, folate, vitamin A (weekly)* *-Not receiving supplements*	In school	12-15 years Female and male Pre and post puberty/menarche	19% (baseline) 30% (population)	Supplement intake & dietary intake: -Iron -Vitamin A		None	No effect on Hb concentration of supplementation with 60 mg iron, 250 µg folate and 10 000 IU vitamin A, either alone or in combination. No evidence on underlying factors for anemia.	Low compliance due to side effects reduced effective duration of supplementation which was likely too short for iron supplementation to improve Hb concentrations; dosage of Vitamin A might have been too low and duration too short for an effect on Hb.
Dillon et al (2005)	Tangerang (rural)	202 (RCT) *-Supplementation* *-16 weeks* *-Iron (weekly)* *-Placebo (weekly)*	In school	11-17 years Female Post-menarche	54% (baseline)	Supplement intake: -Iron		Deworming	Iron supplementation increased concentrations of Hb and serum ferritin. ID contributes to anemia.	Duration of supplementation too short for a long-lasting effect (16 weeks after supplementation). Inflammation not taken into account, so ID may be underestimated
	East Jakarta (urban)	107 (cross-sectional)	In school	15-18 years Female Post-menarche	45% (baseline)	Dietary intake & status markers: -Vitamin B2	NA	None	Vitamin B2 status was positively associated with Hb concentrations and plasma ferritin.	Study design does not allow to infer etiological causation. Inflammation not taken into account, so ID may be underestimated.
	Tangerang (rural) Central Jakarta (urban)	749 (3 cross-sectional studies)	In school	11.3-17.1 years Female Post-menarche	9% (study 1) 31% (study 2) 57% (study 3)	Status markers & supplement intake -Vitamin A -Vitamin B2	NA	Inflammation markers measured	Vitamin A deficiency was related to low Hb concentration (2 of 3 studies and when all studies combined); Vitamin A deficiency was related to low plasma ferritin concentration (all 3 studies). B_2_ status was not related to Hb concentration or plasma ferritin. Not enough evidence to infer role of vitamin A or B2 on iron stores.	For vitamin A, evidence was weak because of low precision in the measurement that was achieved with this sample size. Study design does not allow to infer etiological causation.
	Central Jakarta (urban)	258 (RCT) *-Supplementation* *-8 weeks* *-All groups received iron* *-vitamin A (daily) +* *-Placebo (daily)* *riboflavin (daily) +* *Placebo (daily)* *Vitamin A+ riboflavin (daily)* *-Placebo (daily) +* *-Placebo (daily)*	In school	11-17 years Female Mostly post-menarche	100% (baseline)	Supplement and dietary intake & status markers: -Vitamin A -Vitamin B2 -Iron	-Other micronutrient deficiencies (B6, folic acid, B12, copper) -Hemoglobinopathies such as Thalassemia.	Inflammation markers measured	Lack of conclusive evidence on the contribution of vitamin A to the etiology of anemia. Riboflavin did not meaningfully contribute to improved Hb and iron store levels, and only resulted in no or only a marginal increase in Hb and iron stores above that achieved with iron supplementation alone. Iron status improved as a result of iron supplementation but did not respond to additional supplementation with vitamin A, riboflavin or both.	Lack of conclusive evidence can be attributed to weaknesses in study design, ie, lack of a treatment group with and without vitamin A, or to poor compliance; most girls had mild anemia; therefore, only weak effects could be observed. Degree of upregulation of erythropoiesis may have not been achieved in this study population and conditions necessary for a hematopoietic response to supplementation may be an inadequate status with respect to either of the vitamins.
Kurniawan et al (2006)	Teluk Naga and Kosambi (peri-urban coastal)	133 (cross-sectional)	In school	10-12 years Female Mostly premenarche	2.1% (population) 100% (baseline)	Dietary intake & status markers: -Iron -Protein/energy malnutrition	-Vitamin A -Hemoglobinopathies -Exposure and response to infectious diseases (worm infection, diarrhea, typhoid, otitis and mastoiditis, conjunctivitis, varicella, measles and tuberculosis.	Deworming	Lack of iron supply for Hb formation and inadequate intake of iron rich foods are the main contributing factors for anemia in this study population. Underweight, thin and stunted girls had a higher risk of having IDA. ID was an important contributor to anemia and malnutrition was strongly associated with anemia.	Causation cannot be inferred due to study design; since serum ferritin was not corrected for inflammation, ID based on serum ferritin concentrations may have been underestimated.
Htet et al (2014)	Pramuka island (Jakarta bay area, urban Coastal)	83 (cross-sectional)	In school	Mean age 15.6 ± 1.8 y Female NA	100% (baseline)	Supplement intake & status markers: -Iron -Exposure and response to infectious disease (subclinical inflammation)	-Hemoglobinopathies -Other micronutrient deficiencies	Inflammation markers measured	ID may be an important contributor to anemia. Subclinical inflammation may have been an underlying factor for IDA.	Sample size too small for statistical significance in the difference in hepcidin concentrations between iron-deficient vs non-iron deficient; too short duration of supplementation to reveal significant associations between hepcidin and Hb or SF; subclinical infections during the study may have limited iron absorption. Study design does not allow to conclude that subclinical inflammation might have affected response to iron supplementation. Hepcidin data was not available for all participants, thereby decreasing observed association.
Oy et al (2019)	Malang district, East Java (rural)	355 (cross-sectional)	In school	15-18 years Female Post-menarche	20.2%	Dietary intake: -Iron -Calcium -Folate -Vitamin A	-Energy intake -Other micronutrient deficiencies	Deworming	Iron, calcium, folate, and vitamin A were problem nutrients among anemic girls.	NA
Nurleila et al (2012)	Waitabula, East Indonesia (urban coastal)	3449 (retrospective cross-sectional)	NA	10-15 (subgroup) y Female and male NA	NA	-Malaria	NA	NA	*Plasmodium falciparum and Plasmodium vivax*, along with possible codeterminants, substantially contribute to the burden of anemia.	Possible confounding by endemic coinfections.
Htet et al (2014)	Pramuka island (Jakarta bay area, urban Coastal)	83 (cross-sectional)	In school	Mean age 15.6 ± 1.8 years Female NA	100% (baseline)	Supplement intake & status markers: -Iron -Exposure and response to infectious disease (subclinical inflammation)	-Hemoglobinopathies -Other micronutrient deficiencies	Inflammation markers measured	ID may be an important contributor to anemia. Subclinical inflammation may have been an underlying factor for IDA.	Sample size too small for statistical significance in the difference in hepcidin concentrations between iron-deficient vs non-iron deficient; too short duration of supplementation duration to reveal significant associations between hepcidin and Hb or SF; subclinical infections during the study may have limited iron absorption. Study design does not allow to conclude that subclinical inflammation might have affected response to iron supplementation. Hepcidin data was not available for all participants, thereby decreasing observed association.

Abbreviations: Hb, hemoglobin; ID, iron deficiency; RCT, randomized controlled trial; SF, serum ferritin; NA, not available.

^a^ We define “main etiological determinants assessed” as the determinants outlined in our conceptual framework in Figure 2 and that were explored as being the main contributors to anemia in the study in question.

^b^ We define “other etiological determinants mentioned” as the determinants that are outlined in our conceptual framework in Figure 2 and were not the main contributors to anemia in the study in question but are known to be associated and/or may lead to anemia.

**Table 2.** Studies and Their Findings on Etiological Factors for Anemia Among Adolescents in Indonesia Across the 3 Etiological Categories.

	Kaetelhut et al (1996)	Angeles- Agdeppa et al (1997)	Soekarjo et al (2001)	Februhartanty et al (2002)	Soekarjo et al (2004)	Dillon et al (2005)				Kurniawan et al (2006)	Nurleila et al (2012)	Htet et al (2014)	Oy et al (2019)	Number of studies
	Nutrition													
Iron deficiency	+	+	–	+	0	+	–	–	+	+	–	+	+	8
Vitamin A deficiency	+	–	+	–	0	–	–	+	0	–	–	–	+	4
Folate deficiency	+	–	0	–	–	–	–	–	–	–	–	–	+	2
Vitamin B12 deficiency	–	–	–	–	–	–	–	–	–	–	–	–	–	0
Vitamin C deficiency	+	–	–	–	–	–	–	–	–	–	–	–	–	1
Vitamin B2 deficiency	–	–	–	–	–	–	+	0	0	–	–	–	–	1
Calcium	–	–	–	–	–	–	–	–	–	–	–	–	+	1
Protein/energy malnutrition	–	–	–	–	–	–	–	–	–	+	–	–	–	1
	Genetic hemoglobin disorders													
G6PD	–	–	–	–	–	–	–	–	–	–	–	–	–	0
Red cell membrane defects	–	–	–	–	–	–	–	–	–	–	–	–	–	0
Hemoglobinopathies	–	–	–	–	–	–	–	–	–	–	–	–	–	0
	Infection and inflammation													
Soil-transmitted helminths	–	–	–	–	–	–	–	–	–	–	–	0	–	0
Malaria	–	–	–	–	–	–	–	–	–	–	+	–	–	1
Schistosomiasis	–	–	–	–	–	–	–	–	–	–	–	–	–	0
Tuberculosis	–	–	–	–	–	–	–	–	–	–	–	–	–	0
HIV/AIDS	–	–	–	–	–	–	–	–	–	–	–	–	–	0
Malabsorption & disorders of the small intestine (EED)	–	–	–	–	–	–	–	–	–	–	–	–	–	0
	Menstrual disorders													
Heavy menstrual bleeding	–	–	–	–	–	–	–	–	–	–	–	–	–	0

Abbreviations: EED, environmental enteric dysfunction; G6PD, glucose-6-phosphate dehydrogenase; (0): no association; (+): positive association; (–): no data.

### Nutritional Causes of Anemia

#### Iron deficiency

Lack of iron decreases the formation of Hb, resulting in microcytic anemia. Of 10 retained studies, the vast majority demonstrated an association between ID and anemia among adolescents. In a study by Kaetelhut et al, iron supplementation on a weekly basis for 5 weeks, regardless of the other nutrients (folic acid, vitamin A, and vitamin C) added to the supplement, led to an improvement in Hb concentrations.^3^ Nearly 30% of the sample was anemic at baseline, and after supplementation, the anemia rate reduced to 25.6%.

Angeles-Agdeppa et al^4^ looked at the effect on iron stores by comparing different frequencies (daily vs weekly), duration (3 months), and doses (60 vs 120 mg) of iron supplements with other micronutrients (vitamin A, vitamin C, and folic acid). After 12 weeks of supplementation, anemia prevalence had decreased in all supplemented groups. However, the decrease was only significant in the group supplemented weekly with 60 mg iron. A weekly supplement containing 120 mg iron gave fewer positive results, due to frequent associated side effects. After 12 weeks of supplementation, all groups showed significant improvements in Hb.

In an analysis by Februhartanty et al, the authors investigated the efficacy of 2 different iron supplementation schemes versus placebo for a 16-week period among adolescent girls.^5^ The supplements and placebo were administered in 2 different ways: either weekly or during menstruation. The supplementation contributed to a significant improvement in the iron status of the treatment groups compared to the placebo group. Further, weekly supplementation of iron tablets led to a larger improvement in Hb concentration, when compared with the supplementation of iron tablets only during menstruation. In this study, lack of iron was an underlying factor for low Hb.

Soekarjo and colleagues analyzed factors associated with IDA among adolescents^6^ and allude to low iron intake as an important determinant for anemia. Based on the positive effect on anemia of the high consumption of retinol-rich foods observed, they conclude that retinol intake from foods reflects heme iron intake, given the fact that the former generally contains considerable amounts of heme-iron. As retinol intake was low among anemic participants, this would then equally reflect low heme-iron intake. In a later intervention, Soekarjo and colleagues found no effect on Hb concentration of weekly supplementation with iron, folic acid, and vitamin A, either alone or in combination.^7^ They hypothesize that participants may have overreported compliance rates. As a result, the actual effective duration was likely to be too short for iron supplementation to improve Hb concentrations.^7^

The first study of research by Dillon et al—a randomized controlled trial (RCT) looking at the iron status of Indonesian adolescent schoolgirls after 16 weeks of supplementation, and at 16 weeks after cessation of weekly iron supplementation—showed that, at baseline, the prevalence of anemia, ID, and IDA was 54%, 21%, and 15%, respectively.^8^ Consequently, 28% of anemia cases had ID as an underlying factor. After 16 weeks of supplementation, the iron group’s prevalence of anemia, ID, and IDA decreased to 24%, 9%, and 4%, respectively, thereby showing that supplementation had an effect on anemia prevalence and ID.

In their second study, Dillon et al examined the role of vitamin B2 and explored its status in relation to Hb concentration and iron stores among adolescent schoolgirls. The prevalence of anemia, ID, and IDA was 45%, 25%, and 25%, respectively. As such, 55% of anemia may be due to ID. Although the authors argue that the role of inflammation was probably marginal (very few girls had worm infections, and other infections were uncommon in this population), ID and IDA prevalence may have been underestimated as inflammation was not accounted for.

The fourth study by Dillon et al, an RCT measuring the effects of supplementing vitamin A or vitamin B2, in addition to iron, on Hb and serum ferritin (SF) levels, showed an increase in Hb concentration after 8 weeks of iron supplementation with or without vitamin B2 or vitamin A. This implies that anemia in this population was likely to have been due, at least in part, to ID.^9^ The prevalence of anemia and IDA at baseline was 100% and 56%, respectively. Consequently, 56% of anemia was due to ID.

Kurniawan and colleagues compared the characteristics of girls having either IDA or anemia without ID in 2 subdistricts.^10^ Among the anemic girls enrolled for the study, one-third (27%) had low SF concentrations (<12 mg/L), reflecting depleted iron stores. However, since SF was not corrected for inflammation, ID based on its concentrations may have been underestimated. In addition, more than half of the participants (55%) had serum transferrin receptor concentrations >8.5 mg/L, and 81% showed high zinc protoporphyrin values (>40 mmol/mol heme).^10^ On the other hand, ID based on serum transferrin receptor levels may have been overestimated, as the study was not able to exclude hemoglobinopathies.^11^ Likewise, zinc protoporphyrin may have been elevated by increased erythropoiesis caused by hemoglobinopathies and/or inflammation.^12^ We can cautiously assume that 27% of anemic girls had iron as an underlying cause. In reality, however, the prevalence of anemic girls with iron as an underlying cause may range to 55%, or even higher. The total dietary iron intake among girls with IDA was lower than among girls who had anemia without ID.

Htet et al investigated iron status markers after 12 weeks of iron supplementation among adolescent girls.^13^ At baseline, all 83 girls were anemic, while this decreased to 29 (34.9%) at end line. The study further demonstrates that, after supplementation, 36 (43.4%) of the girls were still iron deficient based on SF concentrations; of them, 11 were no longer anemic. Therefore, among the 29 anemic girls, 25 were iron deficient. We can deduce that 86% of girls still had iron as an underlying cause for anemia. Using Optifood, Oy et al revealed that iron was a problem nutrient for both anemic and nonanemic adolescent girls.^14^

#### Folate deficiency

Folate is important in the development of erythrocytes, and the lack of it results in megaloblastic anemia. In all studies where the investigators included folic acid as an added vitamin in the multinutrient supplement, a separate effect of folic acid on Hb concentrations or anemia status could not be inferred, as all treatment groups received folic acid or because folic acid was combined with other micronutrients. Also, blood folate concentrations were not reported. However, folate was seen as a problem nutrient in the study by Oy et al.^14^

#### Vitamin A deficiency

Lack of vitamin A hampers erythropoiesis and may affect iron metabolism as well. Of the 12 nutrition-related studies obtained via our systematic search, 7 highlighted the role of vitamin A. Most of them reported vitamin A status or the role of vitamin A supplementation in combination with iron supplementation.

In an analysis by Kaetelhut et al, the rise in Hb concentration in the multisupplement group was higher than that in the IFA group among anemic adolescents. Although a clear beneficial effect of the multisupplement could not be observed due to the lack of statistical significance, the authors conclude that supplementation with vitamins (A and C in this case) in addition to iron results in an improvement in Hb concentration. A separate effect of vitamin A on Hb and SF concentrations could not be determined, as vitamin A was combined with other micronutrients.

In the study carried out by Angeles-Agdeppa et al, the investigators included vitamin A in the supplement. After treatment, the prevalence of vitamin A deficiency decreased significantly to 0% in the supplemented groups. As all treatment groups received vitamin A, a separate effect of vitamin A on Hb concentrations or anemia status could not be inferred.

Soekarjo and colleagues observed that girls had a higher chance of being anemic when they had lower vitamin A intake from retinol-rich foods, and higher provitamin A intake from plant sources.^6^ Boys had a lower risk of being anemic when they had a higher retinol intake. In a subsequent school-based intervention by Soekarjo et al, vitamin A supplements were not effective in increasing Hb concentration in both girls and boys. As already mentioned with reference to iron, the authors explain this by pointing to a lack of compliance.^7^

The third study in the article by Dillon et al used data from several cross-sectional studies among adolescent girls to assess the relationship between vitamin A and iron status. Their findings reveal that vitamin A deficiency was related to low Hb concentration and plasma ferritin concentration.^15^ Despite the distinct improvement in iron and vitamin A status, supplementation with vitamin A either did not improve or only marginally improved Hb and iron stores above those achieved with iron supplementation alone.^9^ Alongside iron, calcium, and folate, vitamin A was also seen as a problem nutrient in the study by Oy et al.^14^

#### Vitamin B2

Vitamin B2 (riboflavin) deficiency may also contribute to anemia through its effects on iron metabolism, including a decrease in iron absorption, an increase in intestinal loss of iron or the impairment of iron utilization for Hb synthesis.^16^ In the second study by Dillon et al, vitamin B2 status was positively associated with Hb concentrations and plasma ferritin. As a follow-up, the authors conducted an RCT aimed at measuring the efficacy of supplementation with vitamin B2 among anemic girls, on anemia and indicators of iron status. Participants supplemented with vitamin B2 saw their anemia prevalence reduce to 19%. Nevertheless, riboflavin did not meaningfully contribute to improved Hb and iron store levels and only resulted in no or only a marginal increase in Hb and iron concentrations above that achieved with iron supplementation alone.

Dillon et al’s third study assessed the relationship between vitamin B2 and iron status. Their findings reveal that vitamin B2 status was not related to Hb concentration or plasma ferritin.

The fourth study by Dillon et al showed an 8% increase in plasma ferritin in the vitamin B2 group. Despite the distinct improvement in iron and riboflavin status shown in this study, supplementation with riboflavin either did not improve or only marginally improved Hb and iron stores above those achieved with iron supplementation alone.

#### Vitamin C

Vitamin C enhances nonheme iron absorption by enhancing iron solubility. The study by Februhartanty et al highlights that lower amounts of vitamin C and higher amounts of the iron absorption inhibitors calcium and phytate, in their participants’ daily nutrient intake may have resulted in the low bioavailability of iron present in the body.^5^ Such important findings and confounders must be better understood in the target population, by understanding their diet and the extent to which the level of intake may or may not affect iron bioavailability for the body.

In the intervention study by Kaetelhut et al, described above, vitamin C was added to a weekly supplement containing iron, folic acid, and vitamin A. A separate effect of vitamin C on Hb and SF concentrations could not be determined, as vitamin C was combined with other micronutrients.

#### Protein and energy malnutrition

Only one study touched upon the role of energy deficiency in anemia. Research by Kurniawan et al found that the average energy intake was below the Indonesian RDA in both anemic and IDA adolescents.^10^ Adolescent females with acute malnutrition had a higher risk of having IDA, and approximately 50% of anemic girls were underweight and stunted.^10^ Thin girls had a 5-fold higher risk of suffering from IDA than non-thin participants.

#### Infection and inflammation

Seven of the 13 studies referred to infectious diseases as a contributor to anemia, but only 2 featured infectious diseases as the main focus of their study.

#### Malaria

Nurleila et al conducted a retrospective analysis of patients of various demographic groups, including adolescents aged 10 to 15 years, who were admitted to hospital.^26^ In respect of malaria patients who had *Plasmodium falciparum* and *Plasmodium vivax malaria*, the predominant serious and fatal illness they endured was severe anemia. Severe anemia (n = 266) and altered mental state (n = 137) overwhelmingly dominated the 400 patients with a diagnosis of *P falciparum* who were classified as having a serious illness. The pattern was similar among patients with *P vivax*; among the 199 patients diagnosed, 77 had severe anemia, and 86 had an altered mental state. Despite the study’s limitation in terms of excluding other endemic infectious diseases that may have been the main cause of illness among patients, this does point toward the conclusion that *P falciparum* and *P vivax* substantially contribute to the burden of anemia.

#### Helminth infections

In research by Kurniawan et al, worm infection was likely to be an important cause of anemia given that 1 week after deworming, 44.1% of 238 anemic children (Hb <115 g/L) became nonanemic. Additionally, the fact that 30.1% of girls still had a habit of open defecation reveals the poor water, sanitation, and hygiene conditions and underlines the existing risk of infection.

In a study by Angeles-Agdeppa et al, 34% of a subsample (n = 104) of 363 adolescent girls were found to be infested with *Trichuris trichuria*. As such, the investigators decided to deworm all participants at baseline. The prevalence of low retinol concentrations in the placebo group was reduced from 30.7% to 9.3%. The authors suggest this reduction was most likely and partly due to deworming. No inflammation markers were measured.

Infectious diseases are touched upon in the research by Dillon et al. In their first RCT, the researchers found that among the 132 (of 202) girls who submitted stools, 23% had light ascariasis or trichuriasis, but no hookworms. Consequently, the authors were confident that the group differences in SF concentration measured at 16 weeks and 32 weeks after randomization indicated differences in iron status due to iron supplementation, and not subclinical inflammation from helminth infestation.

In their study looking at vitamin B2 status in relation to Hb concentration, although no hookworm was detected, 9% of the girls had light ascariasis and 14% had light trichuriasis.^22^ Consistent with the earlier study, the authors suggest that subclinical inflammation is unlikely to have led to substantial confounding between vitamin B2 status and Hb concentration. Nonetheless, because inflammation indicators were not measured, one cannot rule out the potential underestimation of the prevalence of ID (25%) and IDA (25%), or the overestimation of the prevalence of girls who reached minimal target iron status (50%).

In their third study, no girls were infected with hookworm, and 11% had either mild ascariasis or mild trichuriasis.^16^ The authors are therefore confident that the group differences in nutrient status observed at the end of the intervention were due to the supplementation with vitamin A and vitamin B2 only and not confounded by infections.

#### Inflammation

In a study by Htet et al, which examined how hepcidin correlates with anemia after 12 weeks of iron supplementation among adolescent girls, a non-negligible number of girls (38%) showed evidence of subclinical inflammation.^13^ However, this subclinical inflammation turned out not to be associated with hepcidin concentration, nor with anemia. Although the study design does not allow causal inferences, the findings do suggest that hepcidin concentrations were higher among those who responded poorly to iron supplementation, as a consequence of increased subclinical inflammation.

#### Genetic Hb disorders

Not one study has explored the role of genetic Hb disorders as a main cause of anemia. Kurniawan and colleagues allude to hemoglobinopathies as a potential cause of the inability of erythrocytes to use iron and highlight the need for screening procedures before the implementation of iron supplementation programs. In the final study by Dillon, α- or β- thalassemia traits are mentioned as possible causes of the remaining unexplained anemia at end line.

#### Menorrhagia

Menorrhagia, also referred to as heavy menstrual bleeding, is defined as measured blood loss in excess of 80 mL per cycle. Its sources of variation are still unclear; however, evidence points toward a hormonal cause among adolescent girls.^43,44^ It is a common menstrual disorder in adolescent girls, and a common cause of IDA. Not one study has quantitatively or qualitatively assessed girls’ menstrual blood loss and its relation to IDA in Indonesia.

#### Mapping the evidence onto a conceptual framework

As laid out in Table 2, our analysis finds that 8 of 13 studies have shown a positive association between ID and anemia; 4 of 13 highlight a potential positive association between vitamin A deficiency and anemia; and 2 of 13 mention the correlation between folate deficiency and anemia. Meanwhile, 5 studies of 13 underscore the different etiological determinants for anemia: one examines the role of malaria, another energy malnutrition; one brings vitamin B2 deficiency to the surface, while another highlights the role of vitamin C deficiency in anemia, and another refers to calcium.

**Figure 2.**
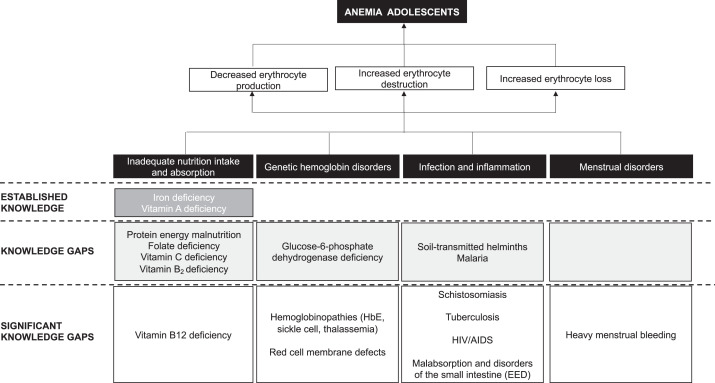
Knowledge gaps on the etiology of anemia among adolescents in Indonesia. Adapted from Figure 3 of a review by Balarajan et al.^45^

Based on these findings, we developed a framework that provides a snapshot of the current etiological knowledge on anemia among adolescent girls and boys in Indonesia (Figure 2). We divide the knowledge into 3 levels, namely (1) significant knowledge gaps, (2) knowledge gaps, and (3) established knowledge. The different etiological factors are categorized in each level.

Based on Table 2 and an eye-ball estimation, we conclude that, in Indonesia, knowledge on genetic Hb disorders and infection/inflammation and menstrual disorders is lagging behind knowledge on nutrition. However, nutrition knowledge on the etiology of anemia among adolescents is mostly limited to iron and vitamin A deficiency, with a data gap on a range of other micronutrients (folate, vitamin C, vitamin B2), and a significant knowledge gap in vitamin B12. With regard to genetic Hb disorders and infection/inflammation, the collected data are too scarce to be characterized as established knowledge.

## Discussion

Based on 6 studies that provided data on both anemia and IDA prevalence, on average 53% to 58% of anemia cases could be explained by ID, whereas 42% to 47% could not, and are caused by other underlying causes (Table 3).^4,8–10,13,22^ Petry et al report an even lower rate of anemia associated with ID in South-East Asia (26.1%).^46^ Although ID seems to be an important cause of anemia in Indonesian adolescents, a similar proportion of anemia cases are due to other causes, and are unlikely to be resolved with iron supplementation.

**Table 3.** Underlying Causes of Anemia Among Adolescents in Indonesia.

Empirical sources	Iron deficiency	Other
Angeles-Agdeppa et al^4^	68%	32%
Dillon et al^8^	28%	72%
Dillon et al^22^	55%	45%
Dillon et al^9^	56%	44%
Kurniawan et al^10^	27%-55%	45%-73%
Htet et al^13^	86%	14%
Average	53%-58%	42%-47%

Any improvement in iron status may be limited when vitamin A status is low. In this review, most studies on vitamin A investigated the combined effect of iron and vitamin A supplementation on anemia. None of them provided conclusive evidence that vitamin A synergistically improved the response of iron supplementation on Hb concentrations. This is despite the reported prevalence of vitamin A deficiency of 7% to 30% in 4 studies which reported on serum retinol concentrations.^3,4,47^ The lack of conclusive evidence can be attributed to weaknesses in study design,^47^ or poor compliance.^3,4^

There is a lack of knowledge on the contributory role of other micronutrient deficiencies in relation to anemia. Although not considered to be a major causal factor of anemia in low- and middle-income countries,^48^ vitamin B2 and vitamin C are included here, as they are revealed to affect iron status (deficiency) among adolescents, thereby potentially reducing anemia. Evidence across the articles on the role of vitamin B2, vitamin C, calcium, and folate is too scarce and heterogenous to lead to any conclusive evidence. It is worth noting, however, that multiple micronutrient supplementation, as compared to iron supplementation alone, appears to have some beneficial effects on Hb concentration.^3,4^ In terms of vitamin B2 status and anemia, 3 of the studies by Dillon et al are somewhat contradictory, since they found that vitamin B2 was positively associated with Hb concentration and plasma ferritin in one study, but not the other 2. However, the RCT did reveal that participants supplemented with vitamin B2 saw their anemia prevalence reduce to 19%, while their Hb and iron store levels did not increase, or only marginally increased above that achieved with iron supplementation alone. These findings lead to inconclusive evidence on the role of vitamin B2. No studies were found on the role of vitamin B12, an essential vitamin for the production of red blood cells, or other B vitamins.

Box 1 Nutritional Anemia in Indonesia.

IDA in Indonesia remains the most significant contributing factor for low public health, with 28.1% of children younger than 5 are affected by it, 29% of 5- to 12-year-olds, 37.1% of pregnant women, and 22.7% of both 13- to 18-year-old female teenagers and women of childbearing age.^1^

Vitamin A has received decent attention, compared to other nutrients. Indonesia began vitamin A supplementation in the 1970s to combat vitamin A deficiency,^17^ essentially eradicated severe “clinical” deficiency, although “subclinical” deficiency is estimated to affect approximately 21% of children in Indonesia.^1^ Additionally, earlier reports suggest that vitamin A deficiency is prevalent among Indonesian women.^18–20^

On the other hand, estimates around folate and B12 deficiencies are scarce. While folate is involved in the synthesis and methylation of DNA, vitamin B12 is an important cofactor in folate metabolism. Dietary inadequacies of these nutrients can lead to impaired DNA synthesis and megaloblastic anemias. During pregnancy, deficiencies of folate and vitamin B12 are associated with a number of negative health outcomes.^21^ Although a few studies suggest that folate status and B12 may be poor, there are very few nationally representative studies available for folate and B12 globally, an observation that is reflected in Indonesia.

Riboflavin deficiency is particularly common in areas where intake of meat and milk/dairy products is low. With the exception of the studies by Dillon et al, there are no data available on the riboflavin intake and status of different population groups in Indonesia.^9,15,22^ Accumulating evidence indicates that suboptimal riboflavin status is a widespread problem across both the developed and the developing world.^23–25^

Protein and energy malnutrition are often linked to anemia, owing to similar root causes. Undernutrition rates in Indonesia are high; 36% of children younger than 5 are stunted, 14% are wasted, and 18% are underweight.^1^ One-third of adolescents aged 16 to 18 years are stunted, with variation across provinces. Thinness affects about 11% of adolescents aged 13 to 15 years, and 9% of adolescents aged 16 to 18 years. Overweight and obesity affect a significant proportion of the population, including about 11% and 7% of adolescents aged 13 to 15 years and 16 to 18 years, respectively.^1^ Although the relationship between weight gain and iron is not fully understood, evidence suggests that obesity is associated with anemia and ID indicators. Further research is needed, as few interventions studies have been designed to address ID among overweight or obese populations.

Abbreviation: ID, iron deficiency.

Unless taken into account, subclinical inflammation can interfere with the assessment of micronutrient status, especially with iron and vitamin A status indicators.^49,50^ Only 3 studies measured inflammatory markers such as C-reactive protein (CRP) and alpha-1-glycoprotein (AGP).^13,47^ Serum ferritin, as a positive acute-phase protein, may increase due to inflammatory reactions, and can conceal any existing ID. Vice versa, serum retinol is transported in the blood stream bound to retinol binding protein, which is a negative phase protein. As such, serum retinol concentrations will fall in the case of inflammation, and true vitamin A deficiency may be overestimated. It is therefore recommended that measures of iron and vitamin A status are adjusted for inflammation.^51,52^ One of the studies showed that 38% of its study population had subclinical inflammation (measured with CRP and AGP), which turned out not to be associated with anemia.^13^ None of the other 10 studies included have made adjustments for inflammation, and therefore reported ID may be underestimated, and vitamin A deficiency may be overestimated. No studies put into evidence the role of overweight/obesity and chronic inflammation with anemia.

Box 2 Exposure and Response to Infectious Disease in Indonesia.

In Indonesia, soil-transmitted helminth infections are persistent, with a prevalence ranging between 20% and 50%, and even higher in some districts.^27^ Only 2.5% of the population live in high-malaria-endemic areas.^28^ A major milestone was reached in 2017, with more than half of all districts officially declared malaria free. Between 2004 and 2014, the country achieved a 17% and 87% overall decrease in malaria cases and deaths, respectively.^29^ However, progress is easily reversible due to human migration from high- to low-transmission areas within the country.^28^ The disease burden and parasite distribution in Indonesia are geographically unequal, with 80% of reported cases between 2005 and 2014 found in the eastern part of the nation, particularly Papua, Western Papua, East Nusa Tenggara, Maluku, and North Maluku. With particular reference to schistosomiasis, *Schistosomiasis japonicum* is the most prevalent species and currently endemic in 3 areas of Central Sulawesi.^30^

According to UNAIDS, Indonesia accounts for nearly a quarter (23%) of AIDS-related deaths, and 18% of new infections in the Asia-Pacific region. About half of new infections in the country are among young people (aged 15-24 years).^31^ Tuberculosis has remained among the top 5 leading causes of DALYs since 1990.^32^ Indonesia experiences intermediate to high hepatitis B virus (HBV) endemicity, and HBV infection prevalence was 7.1% in 2013, compared to 9.4% in 2007.^1,33^ Nevertheless, HBV infection continues to occur during early childhood, as shown by the 5% prevalence of Hepatitis B surface antigen among children younger than 5.^33^ According to Local Health Office data from 5 areas, the percentages of children receiving 3 doses of hepatitis B vaccine are high (73.9%-94.1%), whereas the birth dose coverage is less than 50%.^34^

Indonesia’s coverage with the first dose of measles vaccine has plateaued at 75%, in the midst of the global rise in vaccination hesitancy. Vaccine coverage is at risk without continuous dialogue with key stakeholders and influential decision-makers.^35^

Abbreviations: DALY, disability-adjusted life year; UNAIDS, Joint United Nations Programme on HIV/AIDS.

In relation to the exposure and response to infectious disease, one study suggested that malaria may have been the dominant etiologic agent for severe anemia.^26^ Six studies refer to the light or mild presence of hookworms, ascariasis, or trichuriasis in their study population.^3,4,6,8,9,22^ The majority of the studies accounted for these by the administration of deworming tablets prior to the start of the study, as recommended by Oddo et al.^53^ It is not clear, however, how much these helminth infestations contributed to anemia. No data were found on the independent or combined role of schistosomiasis, tuberculosis, AIDS, EED, and malabsorption and disorders of the small intestine in anemia.

No studies directly related genetic Hb disorders to Hb concentrations or anemia. However, a study that was conducted in the same population as the study by Kurniawan et al found that 39.7% of the girls had hemoglobinopathies.^54^ The diversity of glucose-6-phosphate dehydrogenase (G6PD) deficient variants is significant on the island of Sumba, with Vanua Lava, Viangchan, Chatham variants accounting for 98.5% of the 70 samples genotyped in a study by Satyagraha et al.^42^ This hints toward G6PD deficiency as a potential etiological factor for hemolytic anemia.^55^ There was no evidence on thalassemia or other genetic Hb disorders and their relationship with anemia outcome indicators among this study population. In other South East Asian countries, such as Thailand, thalassemia is one of the main causes of anemia among pregnant women.^56^ In Indonesia, thalassemia ranks fifth in terms of catastrophic diseases and is likely to have an important role in the etiology of anemia.^57^

Box 3 Genetic Hb Disorders in Indonesia and Southeast Asia.

Thalassemia is commonly reported in Indonesia. In 1955, Lie-Injo first reported HbE as the most frequently found abnormality among many ethnic groups in Indonesia, ranging from 2.5% to 13.2%.^36^ In later studies, the reported prevalence varied considerably. It was reported as 9.5% in newborns, and 22% in pregnant women, and ranged between 16% and 60% in athletes. The carrier frequency in some areas was between 6% and 10%, while the pattern of mutation varied widely within each region.^36^ The frequency and type of hemoglobinopathy and thalassemia differs depending on geographical area and ethnic group.^36^ The most frequent mutations to be found are HbE (29%), IVSI-nt5 (19%), and Cd35 (8%). Patients treated in the thalassemia center at Cipto Mangunkusumo Hospital (the national referral hospital in Indonesia) consisted of 50% β-thalassemia, 45% thalassemia HbE, and 5% others.^36^

The most common medical problem associated with glucose-6-phosphate dehydrogenase (G6PD) deficiency is hemolytic anemia. In participants with G6PD deficiency, hemolytic anemia is most often triggered by bacterial or viral infections, or by certain drugs (such as some antibiotics and medications which are used to treat malaria).^37^ Studies have confirmed the presence of G6PD deficiency in Southeast Asia, including several Indonesian islands, but few of them have specifically looked at it as a causal factor for anemia, and even less among adolescents.^38–42^ Ovalocytosis, one of the many red-cell membrane defects, is often found in Indonesia, especially in the eastern part of the country in malaria-endemic areas; however, no studies have looked deeper into this hereditary condition’s prevalence and link to anemia in Indonesia.^36^

Abbreviation: Hb, hemoglobin.

Heavy menstrual bleeding was not mentioned in any studies, despite the evidence that heavy menstrual bleeding can be one of the most important determinants of IDA among adolescent girls.^58^ More research is warranted around menstrual bleeding variability and how it affects IDA.

Our review highlights the need to uncover the differences in the etiology of anemia between the sexes, ages, and puberty stages, given the diverting nutritional needs of adolescent boys and girls upon maturation. Anemia in adolescents should preferably be reported separately, according to sex, puberty stage, and age. Moreover, the lack of information on boys is striking. Last but not least, it is clear that out-of-school adolescents are the most vulnerable and most difficult to reach. Strategies must be sought which are aimed at them.

In conclusion, there is a striking lack of knowledge on the etiology of anemia in Indonesian adolescents. In addition to ID, other nutritional factors, infectious diseases/chronic inflammation, genetic Hb disorders, and heavy menstrual bleeding are likely to play an important role and require further research. To decrease the prevalence of anemia, it may not be sufficient only to focus on an improvement in the effectiveness of the existing IFA supplementation programs. Other etiological factors should also be given urgent consideration.
